# On the bistable zone of milling processes

**DOI:** 10.1098/rsta.2014.0409

**Published:** 2015-09-28

**Authors:** Zoltan Dombovari, Gabor Stepan

**Affiliations:** 1 Department of Applied Mechanics, Budapest University of Technology and Economics, Budapest 1521, Hungary; 2 Dynamics and Control Department, Ideko-IK4, Danobat Group, 20870 Elgoibar, Gipuzkoa, Spain

**Keywords:** delay, time-periodic, milling, torus, bistable, chatter

## Abstract

A modal-based model of milling machine tools subjected to time-periodic nonlinear cutting forces is introduced. The model describes the phenomenon of bistability for certain cutting parameters. In engineering, these parameter domains are referred to as unsafe zones, where steady-state milling may switch to chatter for certain perturbations. In mathematical terms, these are the parameter domains where the periodic solution of the corresponding nonlinear, time-periodic delay differential equation is linearly stable, but its domain of attraction is limited due to the existence of an unstable quasi-periodic solution emerging from a secondary Hopf bifurcation. A semi-numerical method is presented to identify the borders of these bistable zones by tracking the motion of the milling tool edges as they might leave the surface of the workpiece during the cutting operation. This requires the tracking of unstable quasi-periodic solutions and the checking of their grazing to a time-periodic switching surface in the infinite-dimensional phase space. As the parameters of the linear structural behaviour of the tool/machine tool system can be obtained by means of standard modal testing, the developed numerical algorithm provides efficient support for the design of milling processes with quick estimates of those parameter domains where chatter can still appear in spite of setting the parameters into linearly stable domains.

## 1. Introduction

In many aspects, machining of metals is still one of the most important manufacturing technologies nowadays. Its importance is undeniable in the car, energy or air industries. Machining not only forms the parts, but in most cases it gives the final shape to them, which means that the quality of the final product is strongly determined by these cutting operations [1].

Among the abrasive methods, drilling, turning and milling are the most used metal cutting operations. In spite of their sophisticated use in industry, these machining operations still have many unresolved problems as quality and productivity demands increase. The quality requirement means a precise machined surface, which can only be ensured by a vibration-free cutting process. The productivity requirement means large material removal rates, which induces large bites of material and increased rates of motion. In order to increase speed, acceleration has to be increased, which needs lighter design of machine tool structures withstanding less excitation force with no vibrations.

Geometrical accuracy of metal cutting operations depends on the reflected static stiffness of the entire machine at the tip of the tool. This cannot be granted if the operation is subjected to vibrations between the tool tip and the workpiece. By far the most dangerous vibration problem in machining operations is related to the so-called regenerative effect. This arises due to the variation of the chip geometry during cutting: the cutting edges are excited by forces that depend on their vibration history. This was first recognized by Tobias & Fishwick [2] and Tlusty & Spacek [3] in the 1950s. They used the name ‘lobes’ for the shapes of the so-called instability domains in the parameter plane of the spindle speed and the depth of cut, where the regenerative vibrations occur.

The regeneration phenomenon can be modelled by delay differential equations (DDEs), which have quite similar properties to ordinary differential equations but in infinite-dimensional phase spaces, just as partial differential equations do. These equations are part of the family of the so-called functional differential equations [4–6], the theory of which was developed much later than the introduction of the idea of the regenerative effect in machining. From the mathematical point of view, turning and drilling operations are autonomous systems, whereas milling processes are time-periodic non-autonomous systems, the steady-state periodic solutions of which are referred to as stationary cutting/milling. The linear models of milling operations lead to time-periodic parametrically excited DDE governing equations, which can be investigated by the Floquet theory [7,8] extended for infinite-dimensional systems.

For linearized models, a great number of frequency-domain-based as well as time-domain-based methods have been developed in order to determine the asymptotic behaviour of stationary cutting. Generally speaking, the methods in the frequency domain are ready to accept the measured frequency response functions (FRFs) directly, which makes their application convenient in industrial environments [9–12]. Still, these methods have difficulties in including complex nonlinear cutting models and they only serve the critical (non-hyperbolic) borders, from which the actual stability boundaries have to be ascertained; this raises additional difficulties when stable and unstable islands exist in the stability charts. Time-domain-based methods [13–15] are receptive for general cutting models and can present asymptotic properties in any set of technological parameters; however, these methods use extracted modal parameters, which require additional processing of the measured FRFs.

When not only asymptotic behaviour but also large-amplitude vibrations are analysed, the use of nonlinear models is unavoidable. There are many sources of nonlinearities in a real machining environment; however, they can all be neglected compared with the nonlinear and even non-smooth effects originating in the cutting force characteristics. The nonlinear sense of the cutting force can induce complex large vibrations (of the order of magnitude of chip thickness) emerging from corresponding bifurcations of the stationary cutting process. While the suspected bifurcation points can be predicted by means of linear DDE models, the emerging vibrations cannot be identified using linear theories only. From an engineering viewpoint, this becomes a critical issue if the new family of orbits related to the emerging intricate nonlinear vibrations are unstable, and they exist in the linearly stable parameter domains of stationary cutting. Owing to these unstable orbits, stable periodic orbits and stable threshold orbits coexist, that is, stable stationary cutting and persistent chatter coexist in a real machining operation, and only the levels of unpredictable perturbations and uncertainties determine which one will dominate the cutting process. This is the reason why engineers name these parameter regions as uncertain or unsafe zones (UZs) embedded in the stable cutting parameter domains.

Such zones were first identified in the work of Shi & Tobias [16] where a hysteretic behaviour was identified experimentally in a milling process. In this historical experimental result, the authors showed that the stationary cutting process is quite sensitive to external perturbations close to the stability limits but still within the stable region. The same phenomenon was then investigated in turning processes, that is, in autonomous systems using analytical methods, and the subcriticality of the related Hopf bifurcation was identified as a cause of the uncertain behaviour [17–19]. Later, more extensive measurements presented similar phenomena in the case of turning and milling processes [20,21]. Among these results, Stepan *et al*. [21] also used the experimentally detected uncertain/unsafe/bistable parameter domains to critically analyse the existing nonlinear cutting force characteristics models used in industry.

**Figure 1.**
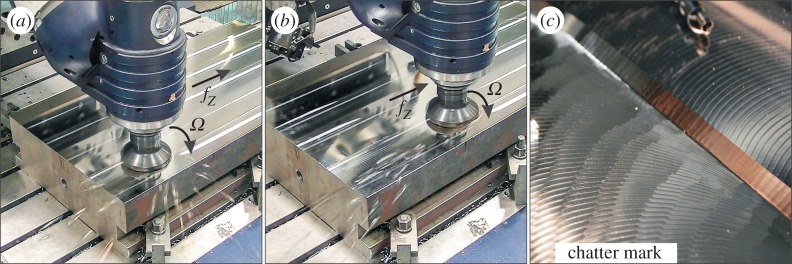
Montage of real case measurements. (*a*) The process is in a chattering state as the cutting edges are leaving the surface of the workpiece: chips are separated in an interrupted way and escape the cutting zone in an irregular manner, leaving a wide sector covered by the traces of the rocketing chips. (*b*) The milling process is in a state of stable stationary cutting, so loss of contact (fly-over) does not appear: chips are separated continuously and fly-away in a regular manner. (*c*) The irregular surface pattern caused by the chattering state is presented.

If the vibrations start increasing during cutting, they soon reach such a level that the cutting edges temporarily leave the workpiece and tend to a threshold vibration that may be a stable periodic, quasi-periodic or chaotic motion [16,22] identified as chatter in the engineering community. This vibration is the cause of irregular patterns on some machined surfaces like the sunflower spirals in turning or thread cutting [23] or the pattern depicted in figure 1*c*. In milling operations, the loss of contact was investigated in various studies, such as [24,25], where milling processes subjected to linear cutting forces were considered, or [26], where highly interrupted but nonlinear cutting was analysed.

The aim of this study is to derive a mechanical model and an algorithm that are suitable to identify the UZs of nonlinear milling processes, where the process is stable for very small perturbations, but still sensitive to increased external perturbations. While the basic phenomenon is expected to be similar to that of the turning process, the time-periodicity and the related parametric excitation that appear in milling processes cause extreme difficulties: the stationary cutting process itself corresponds to a forced periodic motion, and the unstable vibration that emerges at the limit of its stability is already a quasi-periodic one [27]. Clearly, the tracing of this unstable quasi-periodic oscillation till it starts grazing the surface of the workpiece, is a complex task. If found successfully, this also provides estimates for the domains of attraction of the stable stationary milling process within the unsafe cutting parameter zones.

In order to achieve the above aim of this study, the following main results are presented. First, the experimental modal representation of real machine tools is combined with the nonlinear model of the cutting process in §2a. This is a relevant step because experimental modal testing is a linear technique used widely in industry to characterize machine tool dynamics, while the small-scale nonlinearity of the cutting process includes even non-smooth elements and has not been formulated yet mathematically in the case of milling operations due to the time-periodic nature of the process. The corresponding mathematical model is introduced in §2b. Second, a numerical method is developed for tracking unstable quasi-periodic solutions of the overall nonlinear mechanical model of the milling operation, which is presented in §2c. This mathematically challenging task makes it possible to determine the domains of attraction of stable stationary milling. The corresponding estimation of the bistable parameter zones in the stability chart of milling is the main outcome for industrial applications. Thus, the third contribution of this study is the development of a semi-numerical method to determine the borders of the UZs, which is presented and demonstrated in the two industrially realistic representative examples in §3a,b. Also, this work presents an abstract geometric representation of the fly-over (FO) effect [26] or in other words the multiple regenerative effect [25] in milling processes, which is related to the interaction of time-periodic switching surfaces and tori.

The study was triggered by the paper [28], where a stable island was found in the stability chart of milling by means of theoretical methods, which seemed to be impossible to find by experiments or even by time-domain simulations. Finally, careful simulations based on special initial functions managed to ensure stability, but only tiny domains of attraction were perceived. In real case predictions, it is important to know these domains corresponding to such specific stable cutting parameters.

## 2. Nonlinear milling model

As explained in the ‘Introduction’, the dynamic model of the milling tool/machine tool/workpiece system can be assumed linear, so it can be described by conventional experimental modal analysis techniques applied to systems with multiple degrees of freedom (d.f.). In order to compile a realistic nonlinear model of milling processes, the cutting forces have to be characterized primarily as a function of the variation of the chip geometry in the cutting zone. This means that the nonlinear and even non-smooth cutting force characteristics are considered here, which are able to model intermittent cutting. This study does not deal with milling operations performed with intricate tool geometry in the case of variable pitch/helix/lead-angle tools [29–31] or with variable spindle speed [32–34], which further complicate the dynamic model, especially due to the combinations of many different and/or even continuously varying time delays.

**Figure 2.**
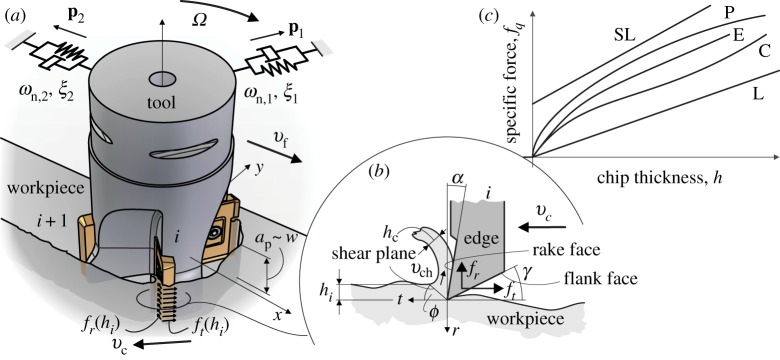
(*a*) Sketch of the milling operation, (*b*) the local representation of the edges and (*c*) the different models of cutting force characteristics. Here, L=linear, SL=shifted linear, P=power, C=cubic and E=exponential cutting force models listed in (2.1).

In engineering practice, it is a widely accepted assumption that the cutting force is linearly proportional to the width *w* of the cut (figure 2). For this reason, the nonlinearity of the resultant force **F** can be described using the specific (local) cutting force **f**=**F**/*w*, which depends on the chip thickness *h* (figure 2*b*) only.

Thus, the core of the cutting force nonlinearity is given by the function **f**(*h*) that is identified empirically by a vast amount of extensive industrial laboratory measurements, all having their own specific advantages and disadvantages. The most relevant specific cutting force functions are summarized one by one in figure 2*c* and given in mathematical form as
2.1
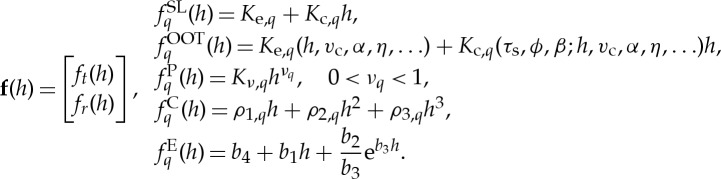
Here, lead angle is considered as *κ*=90° and orthogonality is ensured by zero helix angle *η*=0, which means that the force distribution can be described with the help of its local tangential *t* and radial *r* components in the local (*t*,*r*) edge coordinate system (figure 2*b*). Hence, *q*=*t*,*r* in (2.1).

Among the nonlinear force models, the so-called orthogonal to oblique transformation (OOT) describes the effect of local edge geometry parameters that can introduce some slight nonlinearity [35] via the dependence of the cutting coefficients *K*_c,*q*_ on the chip thickness *h*. The classic power law model (P) was introduced conveniently for linear optimization techniques [36,37]. Polynomial cubic (C) [16] and exponential (E) [38] formulae of cutting force characteristics were used for more accurate nonlinear modelling of cutting forces for specific cutting operations (figure 2*c*).

Considering the simplest milling tool edge geometry with number *Z* of cutting teeth, uniform pitch angle *φ*_*p*_=2*π*/*Z* and, again, zero helix angle *η*=0, the angular edge position can be simply defined by a single angle. Thus, subsequent cutting edge *i* (*i*=1,2,…,*Z*) sweeps through the angular positions *φ*_*k*_(*t*) [39] of a previous cutting edge *k* after a certain regenerative time *τ*_*i*,*k*_, that is
2.2
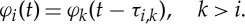
In other words, the angular positions are chained by the delay times *τ*_*i*,*k*_. In the case of large oscillations subjected to the FO effect [26], *τ*_*i*,*k*_=(*k*−*i*)*τ* is an integer multiple of the single constant regenerative delay *τ*=2*π*/*Ω*/*Z* appearing in stable stationary cutting considering constant spindle speed *Ω*.

The actual local chip thickness *h*_*i*_ just cut by the *i*th tooth is expressed as follows:
2.3
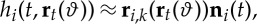
where **r**_*i*,*k*_ is the relative motion of the corresponding *k*th and *i*th edges at angular position *φ*_*i*_(*t*) (2.2), while **n**_*i*_ is the unit vector normal to the plane spanned by the cutting velocity and the actual *i*th cutting edge. The continuous, so-called shift function 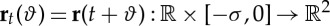 [4,5] describes the planar (*x*,*y*) position vectors of the milling tool centre during the maximal delay *σ* back in time. With all these parameters, the relative cutting edge position can be expressed as
2.4
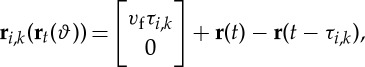
where the secondary motion is defined by the constant feed velocity *v*_f_ (m s^−1^). Note that, in the simplest basic cases, *k*=*i*+1 and *τ*_*i*,*k*_=*τ*.

The resultant cutting force acting on the milling tool can be obtained as the resultant of the overall specific cutting forces integrated in the axial direction along the edge portions that are in cut. As the integration along *z* simplifies to a multiplication with the axial depth of cut *a*_p_ for a straight-fluted milling tool, the time- and delayed state-dependent load on the milling tool is given as
2.5
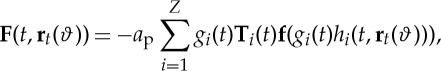
where *g*_*i*_(*t*) is a screen function that switches the specific cutting force off (*g*_*i*_=0) if the edge is not in contact with the workpiece, and switches it on (*g*_*i*_=1) if the edge is in contact. The time-dependent matrix **T**_*i*_(*t*) transforms the specific force between the local system (*t*,*r*) and the spatial system (*x*,*y*) [30].

For a given cutting edge, there are many ways to lose contact during milling. From these cases, the following formula considers two relevant ones:
2.6
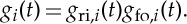
The first component *g*_ri,*i*_(*t*) is the well-known cutter workpiece engagement (CWE) [40], which describes a purely kinematic relationship between the tool and the workpiece considering the feed direction, the radial immersion and the possible helical arrangements of the cutting edges. Since in this work the feed points in the *x*-direction, and the CWE describes a simple milling operation, the tool immersion can simply be described by a constant entry angle *φ*_en_ and a constant exit angle *φ*_ex_.

The second component of the screen function (2.6) *g*_fo,*i*_(*t*) takes into account the so-called FO effect, when the edge leaves the surface of the workpiece due to the vibration of the tool. Contrary to the first kinematic component of the screen function, this one is a purely dynamic effect. In the case of unstable milling processes, it is the FO effect that limits the exponentially growing vibration leading to the large-amplitude stable chatter oscillation.

There are many other possibilities of loss of contact that can be included in the screen function with similar additional multiplicative components. Among these, the so-called missed-cut effects are mentioned here, which appear in the case of uneven edge distances from the rotation axis of the tool. This occurs due to the so-called run-out [41,42] or due to other artificial disturbances on the edge radii as implemented on serrated cutters [30,43].

### (a) Multiple degrees of freedom milling

As explained above, the machine tool structure is considered to be linear and the reflected dynamics can be measured by the techniques of experimental modal analysis using FRFs taken at the tip of the milling tool. As proportional damping occurs in most industrial cases, simple fitting algorithms can identify the vibration modes characterized by their natural frequencies *ω*_*n*,*k*_, damping ratios *ξ*_*k*_ and mass-normalized mode shapes **U**_*k*_=*c*_*k*_**p**_*k*_, *k*=1,…,*m* [44] (figure 2*a*). The mass-normalized modal transformation matrix 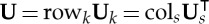 (*s*=*x*,*y*) is formed from the identified mode shapes. In the space of the modal coordinates **q** defined by **r**=**Uq**, the system is represented by the equation
2.7
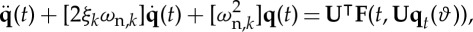
which is a DDE as (2.3) and (2.4) are substituted in (2.5). With the definition of 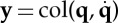, the system is transformed to a first-order DDE in the form
2.8
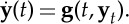
This equation is time-periodic in the sense **g**(*t*,•)=**g**(*t*+*T*,•), where the principal period is *T*=2*π*/*ΩZ*. Consequently, the forced time-periodic stationary solution 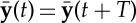 is periodic with the principal period, which is, indeed, a period-one orbit satisfying (2.7).

The period-one orbit of the stationary cutting solution 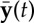 of (2.8) can be found by solving numerically a boundary value problem (BVP). The stable asymptotic behaviour of the period-one orbit 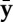 is equivalent to the stable milling process that is desired in machining [9], which also has to meet both productivity [45] and quality requirements [46]. By using perturbation around the stationary cutting solution, the corresponding linear variational system [7] can be determined, resulting in a time-periodic parametrically excited DDE. Combining this with the infinite-dimensional extension of the Floquet theory [7], the criteria of asymptotic stability are to be determined.

The stability criteria for 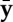 are usually represented in a stability diagram in the parameter plane (*Ω*,*a*_*p*_) of the spindle speed and the axial depth of cut, respectively. The limits of stability are historically called lobes. At these limits, the periodic solution 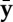 undergoes bifurcations in the corresponding nonlinear system (2.8). These are either secondary Hopf bifurcations, where quasi-periodic vibrations emerge, or flip bifurcations, where period-two vibrations appear. Actually, the flip or period-doubling vibrations occur along stability limits that form closed curves, so they are islands (or lenses, or lentils) rather than lobes, as proved in [47,48].

These different types of stability borders are presented in figure 4*a* for full immersion milling, where *φ*_en_=0 and *φ*_ex_=*π*. The frequency content of the vibrations at the limit of stability are given in figure 4*b* with grey-scale proportional to their strengths. The frequencies belonging to the period-doubling stability limits are at half of the frequency corresponding to the basic time period *T*. The subcriticality of these bifurcations was proved in several special cases such as turning and low radial immersion milling [17,18,20,26], meaning that UZs are likely to appear in the shaded regions of stable stationary cutting.

### (b) The fly-over effect

When FO occurs, the tool leaves the workpiece and its surface remains uncut for the subsequent edge that actually re-enters the workpiece. This means that the surface to be cut is formed by an edge passing through two tooth-pass periods earlier. The time spent between the *k*th and *i*th bites must be an integer multiple of *τ*, that is, *τ*_*i*,*k*_=*r*_*i*_*τ*, where *r*_*i*_ is called the FO index [49].

**Figure 3.**
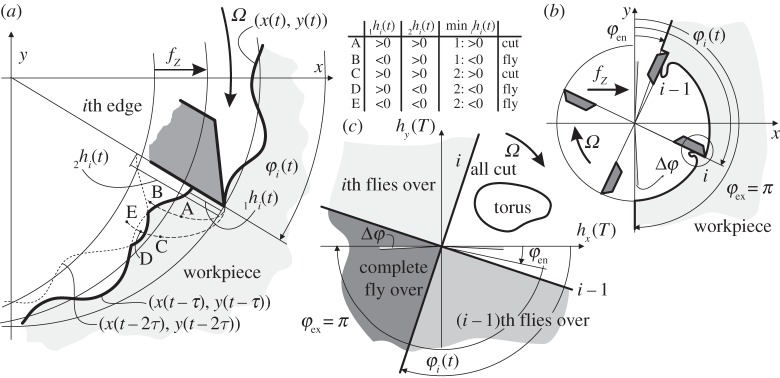
(*a*) A close-up view of *i*th cutting edge of the milling process (_*l*_*h*_*i*_(*t*):= *h*_*i*_(*t*,**q**_*t*_(*ϑ*);*l*)) sketched in panel (*b*), where the edge is about to fly over the surface. In the table, different possibilities for the *i*th edge are listed. (*c*) A possible representation of the time-dependent switching surfaces are shown together with the unstable torus representing a quasi-periodic solution.

Considering the possibilities of these larger delays and the corresponding regeneration effects, the instantaneous chip thickness at the *i*th edge is expressed as
2.9
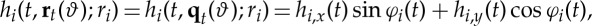
where the corresponding projections of the chip thickness are
2.10
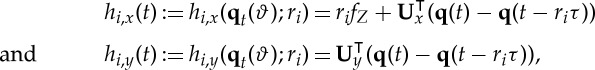
with *f*_*Z*_=*v*_f_*τ* denoting the feed per tooth. The FO index *r*_*i*_ can be calculated as the minimum [50] of all related chip thicknesses possibly cut by the tool up to some reasonable integer number *N*_fo_ (figure 3*a*):
2.11
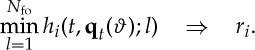

If *h*_*i*_(*t*,**q**_*t*_(*ϑ*);*r*_*i*_)>0, then the *i*th edge cuts the material left by the ((*i*+*r*_*i*_) mod *Z*)th edge; otherwise it flies over. Specifically,

— if *r*_*i*_=1, then no FO is involved prior to the *i*th edge,
— if 1<*r*_*i*_<*Z*, then there is FO prior to the *i*th edge,
— if *r*_*i*_=*Z*, then the *i*th edge cuts the surface cut by itself one full tool rotation earlier, and
— if *r*_*i*_>*Z*, then there is a long FO as the tool loses the regeneration for more than a complete period.

The switches between in-cut dynamics and FO dynamics have a simple geometric interpretation when the trajectories are projected to a specific plane of the otherwise infinite-dimensional phase space that is extended by the *T*-periodic time. FO occurs when the chip thickness seems to be negative, that is, the chip thickness expressed in (2.9) can be reformulated with the FO condition *h*_*i*_(*t*,**q**_*t*_(*ϑ*);*r*_*i*_)<0, yielding,
2.12
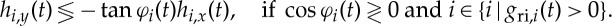
This is represented in figure 3*c*, which shows the momentary switching surfaces in the plane of the chip thickness components frozen at the principal time period *T*, that is, in (*h*_*x*_(*T*),*h*_*y*_(*T*)). The formulated switching surfaces rotate just as the milling tool does in time. For example, in figure 3*c*, when the *i*th edge is at an angular position just overpassing *φ*_*i*_(*t*)=90°, then the corresponding *i*th switching condition is represented with a line a bit inclined from vertical. Consequently, the actual chip thickness *h*_*i*_ has mostly the *x* component affected and the FO condition appears mostly with respect to *h*_*x*_ with ‘*i*th flies over’ region shaded by ‘light grey’ in figure 3*c*. The number of cutting edges is *Z*=4, so the uniform pitch angles are 90°, thus, the next (*i*−1)th edge has a switching surface rotated by 90° in panel (*c*), and the switching condition is represented mostly with respect to *h*_*y*_ component shown by ‘mid-grey’. In the situation presented in figure 3*b*, the (*i*−1)th edge just entered to the workpiece material as 
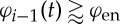
.

The invariant surface covered by the emerging quasi-periodic orbit is a torus, which is represented by a closed curve in this specific projection of the infinite-dimensional phase space in figure 3*c*. This torus can reach those switching condition surfaces and can even overpass them. If all switching conditions are violated, the tool is in complete FO stage (‘dark grey’ in figure 3*c*) and the regeneration is completely switched off due to the lack of the cutting force (2.5), which means that a simple damped oscillator describes the motion of the milling tool until one of its edges bites back again into the material.

As mentioned above, there is an important mechanical/mathematical uncertainty and also a related numerical problem when the edge enters and leaves at *φ*=*π* (see figure 3*b* for the case of down-milling). Physically, it is not clear if the corresponding edge is cutting, rubbing or just missing the cut in these cases due to uncertainties in edge geometry, wear or workpiece material. It is also difficult to follow the conditions defined by (2.12) in these uncertain regions since, depending on the discretization sizes, the switching conditions might easily be violated numerically. For this reason, the switching function component *g*_*ri*,*i*_(*t*) in (2.6) is defined as
2.13
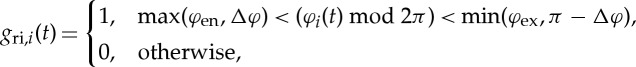
where a sufficiently small Δ*φ* value is chosen where cutting occurs for sure. Clearly, this value has to be introduced at the entering angle, too, where the same problem appears in the case of up-milling.

After identifying the effective momentary chip thickness in (2.11), the component *g*_fo,*i*_(*t*) of the switching function (2.6) can be defined as
2.14
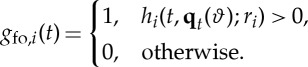


This completes the nonlinear cutting force model (2.5) used in the nonlinear governing equations (2.7) of milling.

### (c) Numerical continuation

In the case of DDEs, it is difficult to find any unstable topological structure by means of time-domain simulation, as one cannot use the standard trick of tracking solutions along reversed time for the saddle-like invariant sets. Similarly to the case of finding the periodic solution of (2.8), semi-numerical BVP solvers [51] are used in order to find the unstable quasi-periodic solutions emerging at secondary Hopf bifurcation points of the identified periodic solutions. More precisely, the BVP solvers determine the topologically invariant skeleton sets on which these solutions exist densely. The identification of this invariant structure is satisfactory to explore the bistable parameter regions.

A specific numerical method has been developed that satisfies the special requirements of fast bistable zone calculations, also to be used in industrial applications. This method includes existing algorithms that determine the linear stability of the stationary solution of general milling processes, then it tracks those invariant tori branches that emerge from secondary Hopf points, and it also follows them along a cutting parameter up to the point where the FO appears. Finally, the numerical method must provide the boundaries of the bistable zones with two-parameter continuations.

To find and to follow the invariant torus branches (ITBs) **u**(*θ*_1_,*θ*_2_) covered by the quasi-periodic stationary solutions 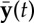 of (2.8), a collocation-based algorithm [52] is implemented using Lagrange polynomials and Chebyshev nodes for collocation. The corresponding invariance equation **G**=**0** with
2.15
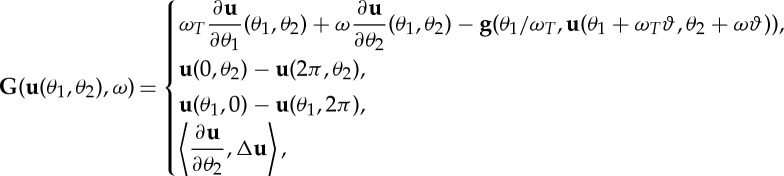
leads to a BVP that can be solved with the Newton–Raphson method (NRM). Note that in (2.15) a single Poincaré phase condition is applied for the unknown frequency *ω* defined over *θ*_2_, as the periodic tooth passing frequency *ω*_*T*_=2*π*/*T* is known for *θ*_1_.

**Figure 4.**
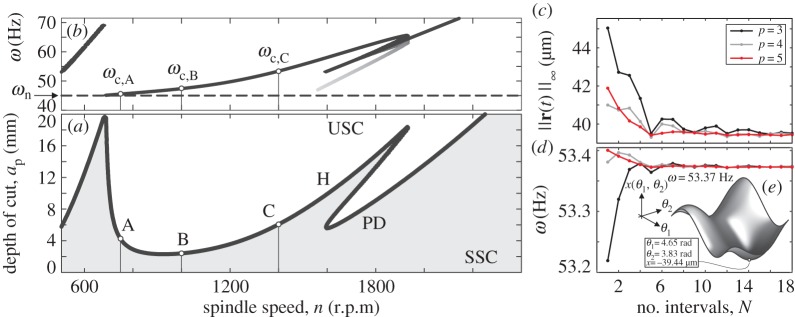
The asymptotic behaviour of time-periodic stationary solution 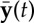. (*a*) The stability chart where secondary Hopf (H) and period-doubling (PD) bifurcation curves are depicted. Stable and unstable stationary cutting are denoted by SSC and USC. (*b*) The basic (chatter) frequency and their harmonics are presented with greyscale proportional to their strengths. (*c*,*d*) The convergence properties of the collocation method implemented for calculating quasi-periodic torus solutions, where different number of intervals *N* and polynomial orders *p* are applied. (*e*) The displacement profile is shown (*N*=20, *p*=5 calculated at *n*=1400 r.p.m., *a*_p_=5.97 mm, table 1).

The obtained numerical solutions were thoroughly compared to the results of finite-difference-based methods [53]. The convergence properties were also analysed as shown in figure 4*c*–*e*. The diagrams with respect to the number *N* of collocation intervals and polynomial order *p* show that the convergence is not monotonic; this phenomenon is mentioned in [53], too.

To find the switching point where the tool leaves the surface, that is, to find the exact boundary of the bistable zone, an additional condition has to be introduced. This requires the use of another free parameter in the extended invariance equation
2.16
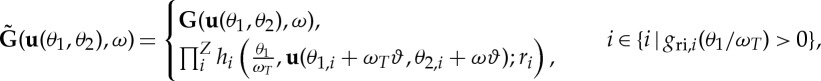
where *θ*_1,*i*_ and *θ*_2,*i*_ are determined from
2.17
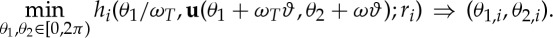
As the FO index *r*_*i*_ is originally time-dependent, it becomes a function of (*θ*_1_,*θ*_2_) in the invariance description. Apart from the non-smooth effect of the radial immersion *g*_ri,*i*_, the implementation of the additional condition in (2.16) can induce abrupt jumps during the use of NRM related to the uncertainty on when (at which (*θ*_1,*i*_,*θ*_2,*i*_)) and at which (*i*th) teeth the FO appears. This causes sharp folds in the continuation, which leads to the breakdown of the method. To bridge these folds, automatic step-size adjustments are applied during the continuation when divergent solutions are experienced.

Finally, in order to extend the calculations of (2.15) and (2.16) for two-parameter continuations, pseudo-arclength predictor–corrector methods [54,55] are implemented.

## 3. Representative examples

One of the simplest possible models was chosen to test the above-described numerical method. The milling tool has *Z*=4 teeth with no helix angle (*η*=0); the cubic force model [16] is applied in (2.1) with the parameters listed in tables 1 and 2.

**Table 1.** Process parameters of 1 d.f. restricted representative example, where *h*_L_=0.15 (mm).

*f*_n_ (Hz)	*ξ* (%)	*k* (N μm^−1^)	**p**	*φ*_en_ (rad)	*φ*_ex_ (rad)
45	4	30	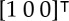	0	*π*

*ρ*_1,*t*_ (N mm^−2^)	*ρ*_2,*t*_ (N mm^−3^)	*ρ*_3,*t*_ (N mm^−4^)	*ν*(1)	*ρ*_*l*,*r*_=*ν* *ρ*_*l*,*t*_	*f*_*Z*_ (mm/tooth)
11.9×10^3^	−161×10^3^	848×10^3^	0.3	*l*=1,2,3	0.1

**Table 2.** Milling process parameters for 2 d.f. case with two relevant structural vibration modes corresponding to figure 7, where *h*_L_=0.25 (mm)

*f*_n_ (Hz)	*ξ* (%)	*k* (N μm^−1^)	**p**	*φ*_en_ (rad)	*φ*_ex_ (rad)
45	4	30	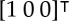	*π*/2	3*π*/4
60	4	30	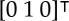		

*ρ*_1,*t*_ (N mm^−2^)	*ρ*_2,*t*_ (N mm^−3^)	*ρ*_3,*t*_ (N mm^−4^)	*ν*(1)	*ρ*_*l*,*r*_=*ν* *ρ*_*l*,*t*_	*f*_*Z*_ (mm/tooth)
14×10^3^	−90×10^3^	200×10^3^	0.3	*l*=1,2,3	0.15

In the first example, only one relevant mode is considered. The direction of this single mode is supposed to coincide with the feed direction *x*. In the second example, two relevant vibration modes are considered, where the directions of these vibration modes are in the perpendicular *x*- and *y*-axes, respectively.

The time-domain simulations were performed by using the dde23 standard solver with a limited force model. The cubic force model was extended by its tangent above a limit chip thickness value *h*_L_ selected outside of the domain of experimental characterization, much above both the used feed per tooth *f*_*Z*_ and the inflection point *h*_inf_=−*ρ*_2,*q*_/(3*ρ*_3,*q*_) values to avoid extremely large cutting forces.

### (a) Restricted one degree of freedom case

If the single vibration mode is in the *x* feed direction, then the regeneration takes the simplest possible representation due to the expression for the momentary chip thickness in (2.9) and (2.10). The modal parameters are taken from the report [28] and summarized in table 1 for the case of full immersion milling.

**Figure 5.**
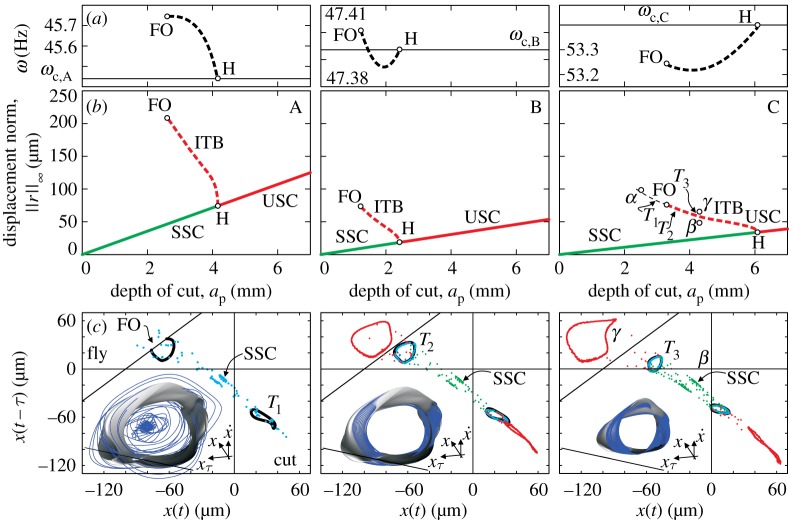
(*b*) The continued ITB that emerge from secondary Hopf bifurcation points (H) separating stable and unstable stationary cutting indicated by SSC and USC, respectively, for the cases belonging to the parameters A, B and C corresponding to points at the Hopf-type limit of stability (figure 4). (*a*) Corresponding frequencies are plotted along the continued ITB up to the point of fly over. (*c*) Planar projections of the tori in the branches are represented at parameter point C for three cases: when FO appears at parameter *T*_1_, at the critical parameter point *T*_2_, and far before the appearance of FO but after the birth of the torus branch at the parameter point *T*_3_. Blue, green and red sets of dots represent time-domain simulations with no perturbations, with slightly larger, and slightly smaller perturbations than the invariant tori. Three-dimensional representations of the tori are presented with the time-domain simulations initiated exactly from the invariant surfaces.

The linear stability limit of the corresponding forced vibration, i.e. the stability boundary of stationary milling, was calculated by means of the semi-discretization (SD) method [8], the corresponding vibration frequencies were determined at the loss of stability and these are depicted in figure 4*a*,*b*. The characteristic multipliers of Floquet theory were also calculated by the SD method, which provided information about the asymptotic stability of the stationary solution 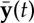. The corresponding stable or unstable stationary cutting is denoted by SSC or USC, respectively, in figures 4 and 5. At the limits of stability, the types of the corresponding bifurcations are also identified by means of the SD method: when the critical characteristic multipliers are complex conjugate as they cross the unit circle of the complex plane, secondary Hopf bifurcations occur; when a critical characteristic multiplier crosses the unit circle at −1, then period-doubling bifurcations occur. In figures 4 and 5, the secondary Hopf and the period-doubling bifurcations are indicated by H and PD, respectively. As the subcriticality of the Hopf bifurcation was analytically proved for the nonlinear autonomous DDE models of turning processes in [17,18], and also the subcriticality of the period-doubling bifurcation was proved for special highly interrupted cutting processes in [56], it was suspected that the numerical results for the nonlinear time-periodic non-autonomous DDE models of milling also present subcritical bifurcations. These numerical results are also confirmed by the experimental observations like the ones in [16,21,57].

Full immersion milling with at least four, equally spaced, even-number cutting edges corresponds to resultant cutting forces that have practically negligible periodicity in time. This explains why engineers rarely experience the PD-type loss of stability in these cases. However, owing to the effect of the nonlinear cutting force, the system is subjected to nonlinearity-induced harmonics that can make the stationary cutting solution unstable through PD. The effect of these harmonics can be followed precisely in the detailed frequency contents in figure 4*b*: unusual harmonic contents appear at spindle speeds around *n*=1700 r.p.m. Also, the *ω*=*ω*_*T*_/2 relationship appears in figure 4*b*. The PD-type stability limits can be distinguished from the H-type limits in experiments by their spectra, although around the intersection of PD and H lobes the vibration frequencies are close to each other. This might cause difficulties during measurements.

The result presented in figure 4 was crucial to provide appropriate initial conditions for the nonlinear invariant equations given in (2.15). The stationary period-one solution 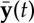 was calculated and pure harmonic vibration was superimposed using a somewhat smaller depth of cut *a*_p_ than the one given by the linear stability limit. This way, the torus branch can be found, although it is saddle-like: owing to the subcritical H bifurcation, the torus is unstable, while it is embedded in an attractive centre manifold. The initial frequency was the principal frequency picked up from the diagram presented in figure 4*b*.

The ITB **u**(*θ*_1_,*θ*_2_) was interpreted in an 11×11 interval phase mesh in (*θ*_1_, *θ*_2_) by using polynomial order *p*=3. This resulted in a 2314-sized nonlinear algebraic equation to solve for the one-parameter continuation case, which was quite robust for initial conditions, unlike the finite-difference method.

In figure 5, the results compiled from the continued solutions are presented to the points where the tool loses contact with the surface of the workpiece at the FO parameter point. The solutions were followed from the initial parameters *n*_A_=750 r.p.m., *n*_B_=1000 r.p.m. and *n*_C_=1400 r.p.m. It is important to emphasize that the norm of the quasi-periodic solution increased less and slower in the higher spindle speed region. Considering a single lobe only, this means that the stationary milling at high spindle speeds is more sensitive to external perturbations than it is in low spindle speed zones (figure 5). The frequency content does not change much during the continuation, although there are slight variations that are probably hardly recognizable in experiments.

At the selected parameter points *T*_1_, *T*_2_ and *T*_3_ along the ITB of the bifurcation diagram in figure 5*c*, the two- and three-dimensional projections of the unstable tori are shown, respectively. In this restricted case, the switching surface *x*(*t*−*τ*)=*x*(*t*)+*f*_*Z*_ is steady in time (2.10). Clearly, *T*_1_ violates the switching condition, *T*_2_ is tangent to it, while *T*_3_ represents a solution when the tool does not leave the workpiece. As the tori presented in figure 5*c*,*d* are finite-dimensional projections of the quasi-periodic solutions embedded in an infinite-dimensional phase space of the corresponding DDE (2.7), virtual self-intersections may appear in certain cases. Note that the invariant tori do not separate the infinite-dimensional phase space into an ‘inner’ and ‘outer’ space. The domains of attraction of the SSC are actually determined by the torus and its infinite-dimensional invariant insets. To show that, time-domain solutions were performed using the invariant torus solutions to create appropriate initial conditions. Then simulations were performed by introducing no perturbation (blue), slightly higher (red) and slightly lower (green) perturbations. Apart from solution *T*_1_, the simulations follow the predicted behaviour. *T*_1_ ‘loses’ solution because it does not exist, most probably due to grazing at the FO point on the ITB.

**Figure 6.**
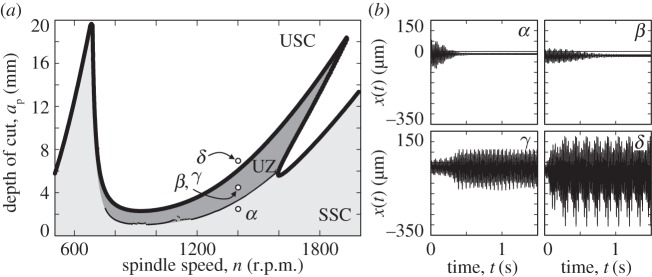
(*a*) The calculated lower boundary of the unsafe (or bistable) zone (UZ), which is coloured dark grey embedded in the grey region of linearly stable stationary cutting. (*b*) Time-domain simulations performed at *n*=1400 r.p.m. at depth of cut levels *α*, *a*_p_=3.3 mm, *β*,*γ*, *a*_p_=4.3 mm and *δ*,*a*_p_=7 mm. The cases *β* and *γ* are distinguished by the initial conditions only (see also *T*_3_ in figure 5*c*).

By means of the extended condition presented in (2.16), the parameters can be found where switching occurs from in-cut to FO. From these points, two-parameter continuation can be initialized in the plane (*n*,*a*_p_) of the linear stability chart. The continuation method worked successfully in a large domain of parameters by using the non-smooth conditions (2.16) directly, although it broke down for cutting speeds *n*∈(850,950) r.p.m. and *n*∈(1100,1200) r.p.m., where the local minimum (*θ*_1,*i*_,*θ*_2,*i*_) related to the local chip thickness (2.17) grazes the non-smooth screen function *g*_ri_ of radial immersion. By means of the automatic increase of continuation steps in these parameter regions, the continuation method successfully got through the critical parts. The results are presented in the form of the dark-grey UZ (or bistable zone) in figure 6*a* together with the results of the time-domain simulations. Apart from *δ*, all time-domain simulations were initialized by a *Π*-magnified version of the invariant torus related to its second frequency. Subsequently, *Π*_*α*_=1.1, *Π*_*β*_=0.99, *Π*_*γ*_=1.01 and *Π*_*δ*_=0 in figure 6*b*. The bistability in the UZ can be recognized in the results of the simulations *β* and *γ*; the corresponding parameter points are also represented in figure 5*c*.

### (b) Multiple degrees of freedom case

**Figure 7.**
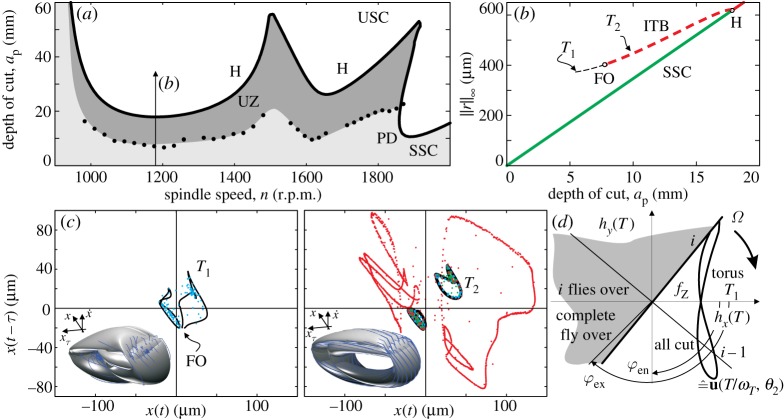
(*a*) Stability of stationary milling for cutting parameters listed in table 2. (*b*) The unstable ITB corresponding to the spindle speed *n*= 1180 r.p.m. also denoted in (*a*) as the axial depth of cut *a*_p_ varies. The ITB includes a critical depth of cut parameter point where fly over appears. (*c*) Unstable quasi-periodic solutions in the form of two- and three-dimensional projections of the corresponding invariant tori along with time-domain simulations (blue, green and red sets of dots are with no perturbations, with slightly larger, and slightly smaller perturbations than the invariant tori). At the parameter point *T*_1_, the effect of FO can already be recognized. The torus is smooth for the parameter point *T*_2_ in which case the tool remains in-cut. The parameter points *T*_1,2_ are shown in (*b*) along the ITB on the two sides of FO. (*d*) The interactions between the switching surfaces and the invariant torus.

In the multiple d.f. case, the FO conditions have more complicated form explained at (2.12) than in the previous restricted 1 d.f. case. The projection of the torus appears in the (*h*_*x*_,*h*_*y*_) plane as a closed curve and the interactions of the time-dependent switching surfaces can be traced. The projection (figure 7*d*) is a composite curve compiled using the modal invariant solutions of the torus **u**(*θ*_1_,*θ*_2_) and the mode shapes **U**. The curve is shifted by the feed per tooth *f*_*Z*_ along the *h*_*x*_ axis. The FO can be identified as it reaches the switching conditions depicted in, for example, the *T*_1_ case in figure 7*d*. As the switching surfaces are rotating in this representation, the unstable quasi-periodic in-cut oscillation might graze these rotating conditions in a disordered manner and then switch to an oscillation that involves FO, too.

In order to avoid sensitive cases of FOs near full immersion milling, the parameter set of an interrupted cutting was chosen in table 2 to demonstrate the calculation of the unstable ITB that is needed for the estimation of the UZ of stationary cutting.

In figure 7*b*, the subcritical secondary Hopf bifurcation is continued along the ITB for the parameters in table 2 at the minimum of the corresponding lobe (figure 7*a*). From the practical point of view, the case presented in figure 7 is important in two aspects: the fairly large stable depth of cut values of SSC are endangered by the UZ in a quite large domain, and also this domain is affected by narrow zones of attraction, which makes it difficult to stabilize the milling process. The ITB was continued until one of the edges left the surface. For the cutting parameters at *T*_1_, the appearing FO ‘scratched’ the otherwise smooth surface of the invariant torus (figure 7*c*). In realistic non-smooth cases, the solution undergoes a grazing bifurcation similarly to the example of B^3^ (big bang bifurcation [58]) presented in [22] for turning, and analogous rapid changes are expected in the ITB. The solution *T*_2_ represents an invariant smooth torus that was only induced by the nonlinear cutting force characteristics during in-cut. This torus and its insets form the domain of attraction of the stable stationary milling process and consequently help to identify the unsafe (or bistable) cutting parameter zone. Time-domain solutions again follow the predicted dynamic behaviour (figure 7*c*).

## 4. Conclusion

We have constructed a nonlinear mathematical model of milling operations that is valid even for large-amplitude tool oscillations when some or all of the cutting edges can leave the workpiece during the self-excited regenerative vibrations. The corresponding time-periodic DDEs include time-periodic non-smooth nonlinearities that are related to the FO effect of the edges. The model is constructed in such a way that it includes the results of experimental modal testing used in industry for characterizing machine tool dynamics.

The stationary milling processes lose stability at cutting parameters presented in stability charts, and essential parts of these stability boundaries are responsible for subcritical secondary Hopf bifurcations. This means that a saddle-like unstable torus emerges at loss of stability, which corresponds to an unstable quasi-periodic regenerative oscillation of the milling tool. We have developed a numerical method that is able to track the corresponding unstable invariant set in the parameter domain where the stationary milling is stable. In some sense, this set separates the stable stationary cutting and the stable large-amplitude regenerative chatter. The corresponding parameter domain is the so-called bistable zone, where stable stationary milling is ‘not safe’, that is, large enough perturbations may lead to (stable) chatter.

The size of this bistable zone is vital information for the design of milling processes. We have constructed a two-parameter continuation method that is able to calculate those cutting parameters—like the critical axial depth of cuts at given cutting speeds—where the switching conditions for the appearance of the FO effect are violated, that is, where the unstable invariant torus grazes the time-periodic switching surface in the infinite-dimensional phase space.
